# Cytocompatibility Evaluation of a Novel Series of PEG-Functionalized Lactide-Caprolactone Copolymer Biomaterials for Cardiovascular Applications

**DOI:** 10.3389/fbioe.2020.00991

**Published:** 2020-08-13

**Authors:** Sandra Pacharra, Seán McMahon, Patrick Duffy, Pooja Basnett, Wenfa Yu, Sabine Seisel, Ulrik Stervbo, Nina Babel, Ipsita Roy, Richard Viebahn, Wenxin Wang, Jochen Salber

**Affiliations:** ^1^Salber Laboratory, Centre for Clinical Research, Department of Experimental Surgery, Ruhr-Universität Bochum, Bochum, Germany; ^2^Laboratory A, Synergy Centre, Ashland Specialties Ireland Ltd., Dublin, Ireland; ^3^School of Life Sciences, College of Liberal Arts and Sciences, University of Westminster, London, United Kingdom; ^4^Rosenhahn Group, Faculty of Chemistry and Biochemistry, Analytical Chemistry – Biointerfaces, Ruhr-Universität Bochum, Bochum, Germany; ^5^Faculty of Chemistry and Biochemistry, Analytical Chemistry – Center for Electrochemical Sciences, Ruhr-Universität Bochum, Bochum, Germany; ^6^Centre for Translational Medicine, Medical Department I, Marien Hospital Herne, University Hospital of the Ruhr-University Bochum, Herne, Germany; ^7^Roy Group, Kroto Innovation Centre, Department of Materials Science and Engineering, University of Sheffield, Sheffield, United Kingdom; ^8^Department of Surgery, Universitätsklinikum Knappschaftskrankenhaus Bochum GmbH, Bochum, Germany; ^9^The Charles Institute of Dermatology, School of Medicine and Medical Science, University College Dublin, Dublin, Ireland

**Keywords:** poly-L-lactide, poly-ε-caprolactone, polyethylene glycol, bioresorbable, cytocompatibility

## Abstract

Although the use of bioresorbable materials in stent production is thought to improve long-term safety compared to their durable counterparts, a recent FDA report on the 2-year follow-up of the first FDA-approved bioresorbable vascular stent showed an increased occurrence of major adverse cardiac events and thrombosis in comparison to the metallic control. In order to overcome the issues of first generation bioresorbable polymers, a series of polyethylene glycol-functionalized poly-L-lactide-co-ε-caprolactone copolymers with varying lactide-to-caprolactone content is developed using a novel one-step PEG-functionalization and copolymerization strategy. This approach represents a new facile way toward surface enhancement for cellular interaction, which is shown by screening these materials regarding their cyto- and hemocompatibility in terms of cytotoxicity, hemolysis, platelet adhesion, leucocyte activation and endothelial cell adhesion. By varying the lactide-to-caprolactone polymer composition, it is possible to gradually affect endothelial and platelet adhesion which allows fine-tuning of the biological response based on polymer chemistry. All polymers developed were non-cytotoxic, had acceptable leucocyte activation levels and presented non-hemolytic (<2% hemolysis rate) behavior except for PLCL-PEG 55:45 which presented hemolysis rate of 2.5% ± 0.5. Water contact angles were reduced in the polymers containing PEG functionalization (PLLA-PEG: 69.8° ± 2.3, PCL-PEG: 61.2° ± 7.5) versus those without (PLLA: 79.5° ± 3.2, PCL: 76.4° ± 10.2) while the materials PCL-PEG550, PLCL-PEG550 90:10 and PLCL-PEG550 70:30 demonstrated best endothelial cell adhesion. PLLA-PEG550 and PLCL-PEG550 70:30 presented as best candidates for cardiovascular implant use from a cytocompatibility perspective across the spectrum of testing completed. Altogether, these polymers are excellent innovative materials suited for an application in stent manufacture due to the ease in translation of this one-step synthesis strategy to device production and their excellent *in vitro* cyto- and hemocompatibility.

## Introduction

Stents are used in percutaneous coronary intervention to restore blood flow in patients with coronary artery disease (CAD) which is characterized by the occlusion of a coronary artery due to plaque formation (Garg and Serruys, 2010; Li et al., 2010). Researchers are dedicated to the development of new biocompatible materials for use in stent devices, that on the one hand provide enough strength for vessel support during constrictive re-modeling while also allowing for good cyto- and hemocompatibility (Van Oeveren et al., 1999; Williams, 2008; Huang et al., 2014; Zhang et al., 2014; Blok et al., 2016). Complications still arise from stent placement, the main issues being in-stent restenosis and stent thrombosis resulting from inadequate mechanical properties or poor biocompatibility (Zhang et al., 2014). Drug Eluting Stents (DES) were successful in reducing the rate of in-stent restenosis (Khan et al., 2012), however, the requirement of longer dual antiplatelet therapy (DAPT) to prevent stent thrombosis means that they are not suitable for all patients. In addition, complications have arisen relating to DES with reported cases of late thrombosis, hypersensitivity and delayed healing (Al-Dehneh et al., 2010). A major disadvantage of BMS and DES stent devices is the permanence of the scaffold associated with adverse events including stent jailing, side vessel obstruction, impairment of vessel geometries and the need for long term anti-coagulant therapy (Waksman, 2006; Meraj et al., 2015).

Fully absorbable stent technologies are the freshest development in this sector and introduce a new paradigm in CAD treatment (Tenekecioglu et al., 2016). Scaffold resorption after vessel repair is thought to offer several advantages including a reduced risk of late stent thrombosis, facilitation of repeat treatments, restoration of vasomotion and freedom from side branch obstruction (Ormiston and Serruys, 2009). Realization has been hampered by material performance inferiority in comparison to permanent metallic counterparts. Two-year follow–up on patients treated with the Abbott ABSORB BVS stent showed an 11% rate of major adverse cardiac events (e.g., cardiac death, heart attack, additional procedures) in comparison to a 7.9% rate for patients treated with the metallic XIENCE drug eluting stent. Furthermore, an increased rate of thrombus formation was observed with the BVS compared to the XIENCE stent (1.9% in BVS versus 0.8% in XIENCE) (Maisel, 2017). The current state of the art absorbable polymer stent technologies (including ABSORB) rely on the polymer poly–L–lactide (PLLA) (Gupta et al., 2007; Iqbal et al., 2013; Verheye et al., 2014), which displays a relatively high elastic modulus with a low elongation at break. PLLA’s high crystallinity and glass transition temperature (T_g_ of 60°C) result in relatively brittle behavior in medical application (Fernández et al., 2012). Furthermore, polymers used in bioresorbable stent (BRS) technologies thus far have presented considerably lower ultimate tensile strengths (UTS) with respect to gold standard metals. Thus, the application is hampered by fracture risks, strut thickness limitations, recoil, deployment limitations and limited expansion among a host of new challenges arising with degradable devices including degradation profile, biocompatibility of by-products, shelf-life concerns, and reduction in mechanical strength as degradation proceeds (Khamis et al., 2014; Collet et al., 2016; Foin et al., 2016; McMahon et al., 2018).

Copolymers offer a potential solution to address many of the homo-polymer performance limitations in absorbable stent devices without significantly compromising relevant material properties that make PLLA such a useful candidate. Copolymerizing of lactide with small amounts of ε-caprolactone (Fernández et al., 2012) yielded a reduced T_g_ and crystallinity, thus improving the elongation at break and presenting a valuable performance tuning opportunity (Ugartemendia et al., 2015). In addition, polyethylene glycol functionalization offers potential to tailor the surface properties in terms of hydrophilicity and therefore control cellular adhesion and responses. PEG grafting techniques are typically multi-step approaches (Kingshott and Griesser, 1999; Lieb et al., 2003; Shin et al., 2009; Vasilev et al., 2010; Melchiorri et al., 2013) which are difficult to translate to the complex architectural design of stents post fabrication. Furthermore, there are significant challenges in stabilizing PEG grafts through the processes of crimping, sterilization, storage and expansion of a stent. With polymer-based stents, there is a new opportunity to solve these issues via incorporation of PEG in the raw materials chemical structure adding significant application potential and by-passing complex grafting procedures (Kim et al., 2011; McMahon et al., 2016). Short PEG end chains (Mw 550 Daltons) were carefully selected to ensure bioresorption of this PLCL solution is not compromised since low molar mass PEG may be eliminated from the body safely (Jeong et al., 2004; Webster et al., 2007).

Combining the two strategies (PEG copolymerization with PLCL copolymers) we have systematically created polymer series that allows refined control over a wider degree of material performance which will become an important biomedical device strategy in future. These polymers have been synthesized using a proprietary approach yielding a cleaner polymer lacking solvent residues therefore inherently reducing the risk of adverse inflammatory effects on the patient and removing the risk of solvent residue interactions with active pharmaceutical ingredients or therapy (Barnes and Harris, 2008).

Here, we present the results of *in vitro* biocompatibility assessment of these new chemically defined lactide/caprolactone/PEG copolymers using a panel of five cell-based tests. While *in vitro* biocompatibility evaluation of medical devices is described by a series of standards collectively under the ISO 10993 set, it does not include available assays that may be more relevant to a cardiovascular application. Therefore, we selected a panel of five *in vitro* tests to easily screen and thoroughly evaluate the cyto- and hemocompatibility of the new materials. On the one hand, we performed the tests required by ISO 10993-5 standard comprising material toxicity testing according to the multiplex assay format and hemolysis testing (ISO 10993-4) (Beuth Publishing, 2009, 2017). In addition, we analyzed the materials’ thrombogenic and inflammation inducing properties. Since ECs maintain a non-thrombogenic blood-tissue interface, regulate thrombosis, thrombolysis, vascular tone and blood flow in their basal state they are considered the ideal non-thrombogenic and non-inflammatory surface (Galley and Webster, 2004). We analyzed EC adhesion to the materials to assess their usefulness in facilitating fast and complete reendothelialization. Platelet adhesion and morphology was used to analyze thrombus formation risk and a leucocyte activation assay based on PMN elastase release was used to test for inflammatory induction. Together, these tests allowed a more in-depth view on all relevant properties of a cardiovascular device material which ideally should be non-toxic, non-hemolytic, non-thrombogenic, non-inflammatory and facilitate EC adhesion.

## Results

### Polymers

#### Synthesis

**FIGURE 1:**
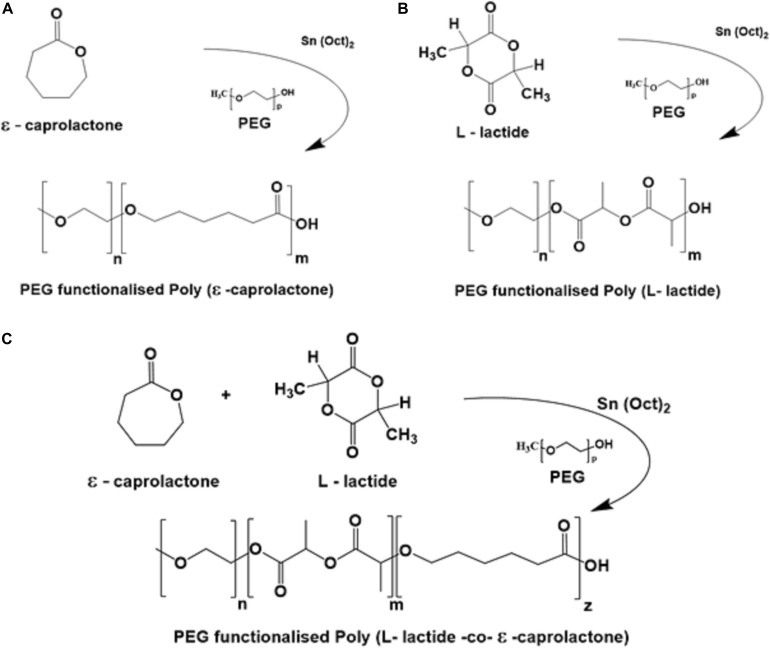
Structural overview on each reaction for the synthesized PEG functionalized materials **(A)** PCL-PEG, **(B)** PLLA-PEG, and **(C)** PLCL-PEG.

PEG-functionalized PLCL polymers of varying composition (lactide to caprolactone of 90:10, 80:20, 70:30 and 55:45, see Figure 1 for synthesis scheme and Supplementary Table S1 for a list of all polymers used in this study) were synthesized using a proprietary polymerization approach. Molecular weight analysis by gel permeation chromatography (GPC) showed that all polymers presented medium to high relative molecular weights and relatively low polydispersity (PDi range of 1.5–2.4, see Table 1).

**TABLE 1:** Results summary of GPC and ^1^H-NMR analyses of synthetic polymers.

**Material**	**M_n_ /kDa**	**M_w_ /kDa**	**PDi^a)^**	**LA**	**CL**	**PLA**	**PCL**	**PEG**
PLLA	374.2	584.6	1.52	0.99	–	1.0	–	–
PLLA-PEG550	236.6	347.8	1.47	0.98	–	1.0	–	+
PCL	61.6	115.0	1.87	–	0.99	–	1.0	–
PCL-PEG550	77.8	175.4	2.26	–	0.99	–	1.0	+
PLCL-PEG550 90:10	116.7	279.8	2.40	0.99	0.99	0.88	0.12	+
PLCL-PEG550 80:20	91.0	164.5	1.81	0.99	0.98	0.80	0.20	+
PLCL-PEG550 70:30	101.3	177.3	1.75	0.99	0.96	0.68	0.32	+
PLCL-PEG550 55:45	83.4	139.8	1.68	0.99	0.99	0.56	0.44	+

*Polymer number average molecular weight (M_*n*_) and weight average molecular weight (M_*w*_) were determined by GPC while polymer monomer purity (lactide: LA and caprolactone: CL), copolymer molar fractions (polylactide: PLA and polycaprolactone: PCL) and presence of PEG were confirmed by ^1^H-NMR. ^*a)*^Polydispersity index = M_*w*_/M_*n*_; The values for M_*n*_ and M_*w*_ resulted from individual determinations.*

The molecular weights were large enough to exceed a critical value for entanglement formation thus allowing comparison across the series. Relative fractions of lactide to caprolactone were confirmed by ^1^H-NMR to lie within an acceptable ± 2 molar fraction of the target molar ratios while polymer purity with respect to monomer residues was ≥98% for lactide monomer conversion and ≥96% for caprolactone monomer conversion (see Table 1). Furthermore, presence of PEG was confirmed by the appearance of a peak at a chemical shift of 3.65 ppm in all PEG-functionalized polymers.

#### Surface Properties of Polymer Films

For the analysis of *in vitro* biocompatibility films of PEG-functionalized polymers and non-functionalized controls were prepared and their surface properties examined via water contact angle (WCA) measurement, FTIR-ATR and SEM (see Table 2 for a summary on all film surface results).

**TABLE 2:** Summary of film surface properties.

**Material**	**Contact angle^a)^**	**PLA^b)^**	**PCL^b)^**	**Crystalline^b)^**	**SEM^c)^**
PLLA	79.5° ± 3.2	+	–	No	–
PLLA-PEG550	69.8° ± 2.3	+	–	No	Flat and smooth
PCL	76.4° ± 10.2	–	+	Yes	Spherulitic structure
PCL-PEG550	61.2° ± 7.5	–	+	Yes	Spherulitic structure
PLCL-PEG550 90:10	65.5° ± 4.1	+	+	No	Flat but roughened
PLCL-PEG550 80:20	67.2° ± 3.1	+	+	No	Alternating smooth and roughened areas
PLCL-PEG550 70:30	75.5° ± 2.0	+	+	Yes	Flat and smooth
PLCL-PEG550 55:45	72.3° ± 6.1	+	+	Yes	Homogeneous ridged structure

**FIGURE 2:**
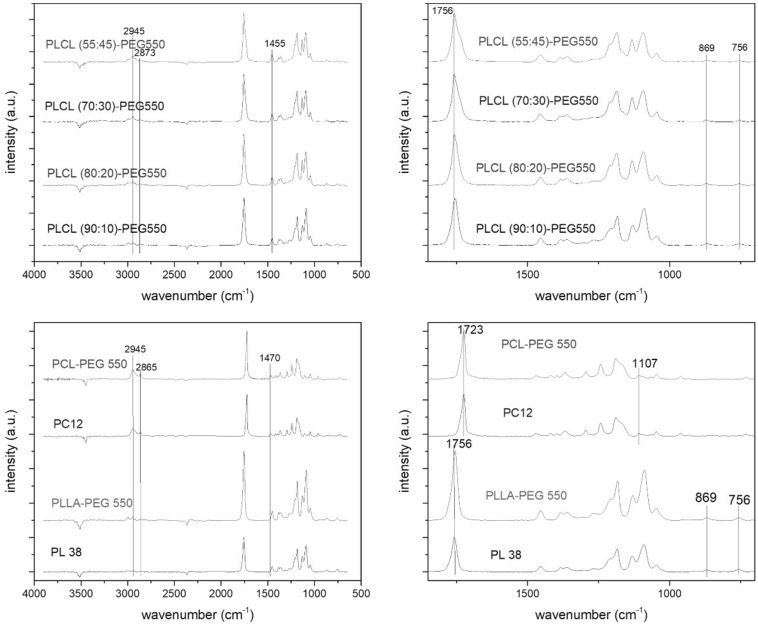
FTIR-ATR spectra of PCL (PC12) and PCL-PEG550, PLLA (PL38) and PLLA-PEG550 and the PLCL-PEG550 series. The figures show overview spectra **(left side)** and zoom-in spectra **(right side)** of the 650 – 1800 cm^– 1^ wavenumber region.

**FIGURE 3:**
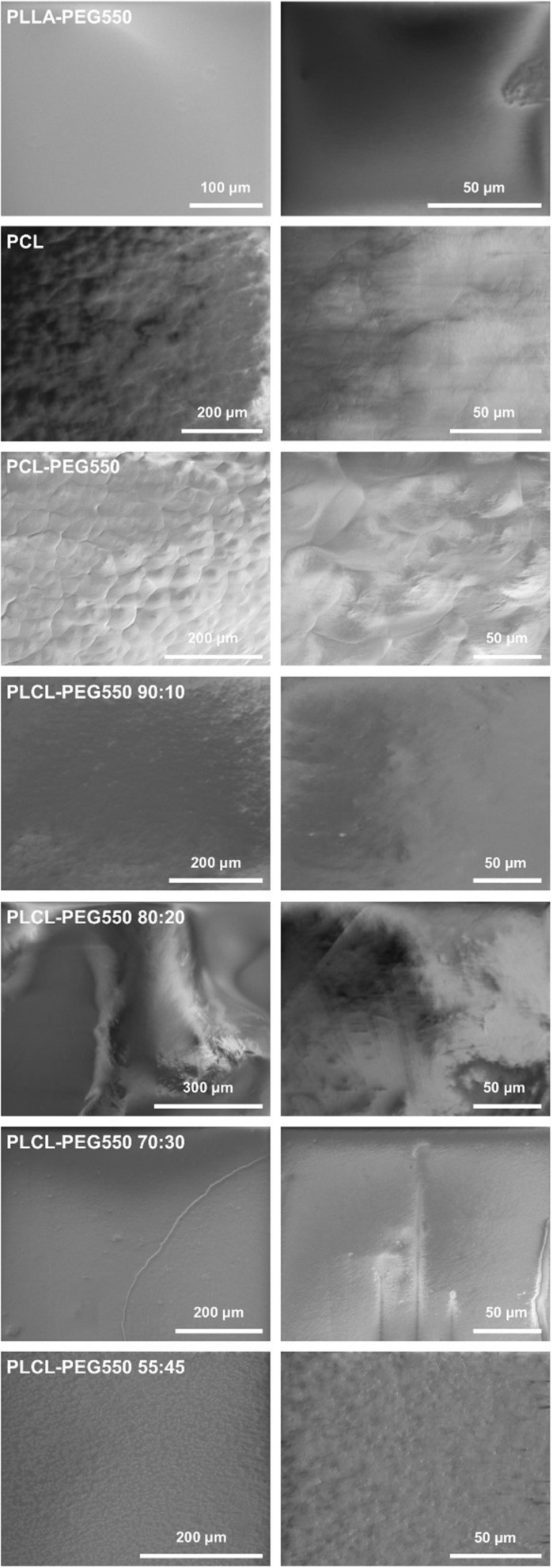
Representative SEM pictures of material films in different magnifications.

*Water contact angles, presence of polylactide(PLA) and polycaprolactone (PCL) as determined by FTIR, FTIR evidence of crystalline surface and SEM appearance are presented. ^*a)*^see Supplementary Figure S2; ^*b)*^see Figure 2 for FTIR graphs and Supplementary Tables S3–S5 for FTIR peak summary; ^*c)*^see Figure 3 for SEM micrographs.*

Water contact angle analyses were conducted in order to get a first impression on film surface characteristics, especially hydrophilicity (see Supplementary Figure S2). Films of the controls PLLA and PCL exhibited contact angles of 80 ± 3° and 76 ± 10°, respectively while incorporation of PEG resulted in a significant reduction of contact angles for the PLLA-PEG550 and PCL-PEG550 films to 70 ± 2° and 61 ± 8°, respectively. Contact angles of the PLCL-PEG series films were lower than non-PEGylated controls while a slight but significant increase was observed with increasing caprolactone content from PLCL-PEG550 90:10 and 80:20 to 70:30 and 55:45.

Polymer film surfaces were analyzed by FTIR-ATR measurement (Figure 2) which clearly proved the presence of lactide and/or caprolactone in the films. The most prominent indicator of PCL presence was the band at 2945 cm^–1^ (asymmetrical stretching of CH_2_, see details on PCL-specific signals in Supplementary Table S3) while in case of PLLA a distinctive signal pattern was observed in the range from 1250 to 1000 cm^–1^ (particularly the symmetrical stretching mode C-CH_3_ at 1045 cm^–1^, see details on PLA-specific signals in Supplementary Table S4) (Schrader, 1995; Oliveira et al., 2013). Using these signals presence of polylactide could be shown in PLLA, PLLA-PEG550 and all candidates of the PLCL-PEG series while caprolactone presence was observed in PCL, PCL-PEG and the PLCL-PEG series. Here, the normalized signal intensities of the caprolactone specific 2945 cm^–1^ signal actually increased with increasing content of caprolactone in the polymer, while the main PLLA signal decreased. The stretching vibration of the carbonyl group was observed as a prominent peak at 1756 cm^–1^ for PLA containing polymers and at 1723 cm^–1^ for PCL. In the mixed polymers, the band at 1723 cm^–1^ for PCL could only be observed as a shoulder. A shift to lower wavelength also is indicative of increased crystallinity which is the case in PCL and PCL-PEG550. Also, in the two polymers with higher caprolactone content (PLCL-PEG70:30 and 55:45) the shoulder to lower wavelength indicates crystallinity which was more pronounced in PLCL-PEG550 55:45. Presence of PEG could not be shown using this method since PEG signals are overlapping with PLA and PCL signals (see details on PEG-specific signals in Supplementary Table S5).

SEM revealed a good surface quality for all polymer films as shown in Figure 3. In all cases, the films were flat and well-formed without presence of air bubbles or holes, whereas these polymers presented differences in terms of surface morphology and topography. Since all film casting was conducted using the same approach, changes in surface morphology can be accredited to the variance in polymer physical properties. In case of PLLA-PEG550, we observed a very smooth surface with no irregularity in terms of surface structuring. PLCL-PEG550 90:10, PLCL-PEG550 80:20, and PLCL-PEG550 70:30 were similar in surface quality showing mild deviation from the flat surface observed in PLLA-PEG550 although PLCL-PEG550 90:10 exhibited a slightly roughened surface. All of these polymers had large lactide fractions leading to relatively similar film formation and surface behavior. Whereas the PLCL-PEG 55:45 material yielded films that presented a more ordered surface morphology with slight elevations created in the form of rising ridges of approximately 1 μm diameter. This can be accredited to the highly elastomeric behavior of this polymer allowing it the flexibility to form these ridges (Fernández et al., 2012). PCL showed a very ordered surface morphology with obvious surface patterns of crystalline areas. PCL-PEG550 was the most crystalline, where the film surface presented spherulite structures.

### *In vitro* Cell Compatibility

Films of PLCL-PEG copolymers were evaluated using a panel of different *in vitro* methods – according to ISO 10993 and beyond. Here, we were specifically interested in the effect the new materials had on cells present at a stent implantation site (especially different types of blood cells and endothelial cells).

#### Toxicity Testing With L929

**FIGURE 4:**
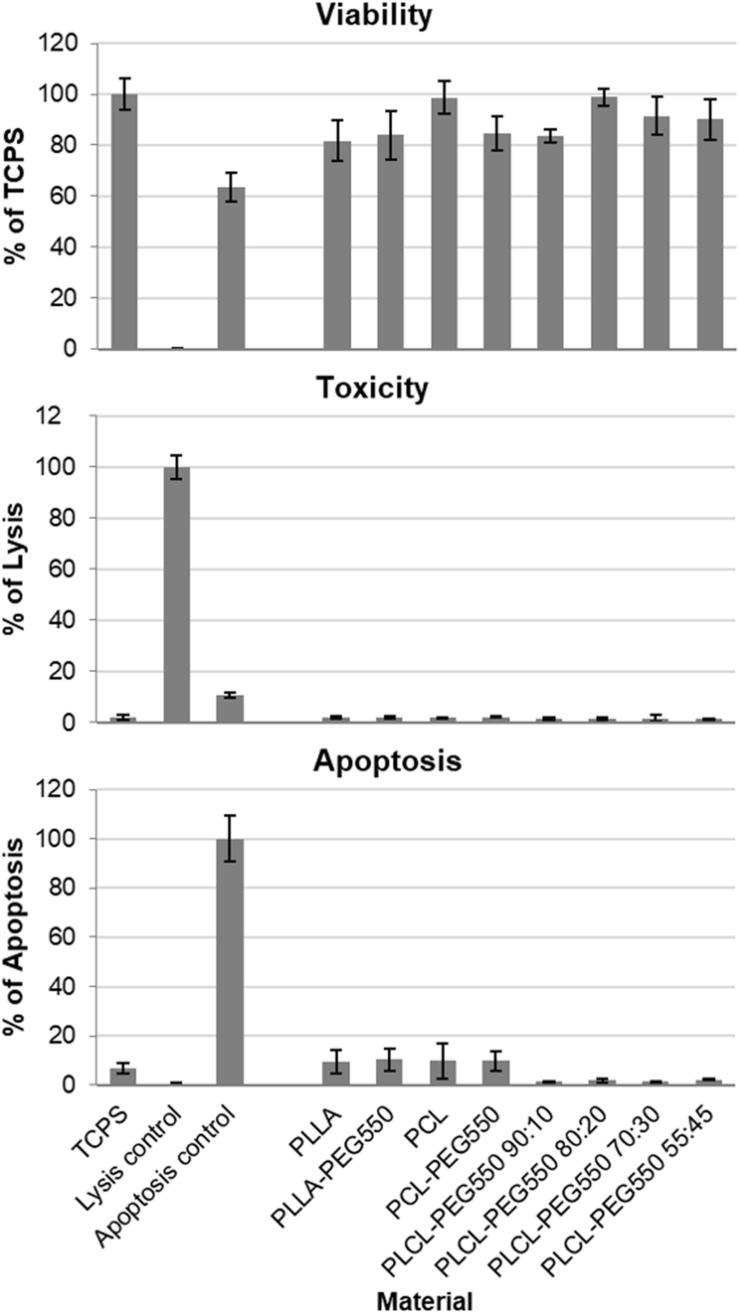
Lactide/caprolactone film cytocompatibility was analyzed in multiplex format using L929 cell line. L929 cells were seeded directly onto material disks and grown for 24 h. Cell viability of attached cells was measured using CellTiter-Blue assay and results are depicted in % compared to TCPS. Cytotoxicity was analyzed using CytoTox-ONE assay, whereby lysed cells were used as positive control (100% of cell lysis) and apoptosis induction was determined using ApoONE assay, by using staurosporine induced cells as positive control (100% of apoptosis). Measurements were carried out in quadruplicate.

First of all, we tested if the materials caused any adverse reactions in a standard cell line as suggested in ISO 10993-5 (Beuth Publishing, 2009). Since finally, cardiovascular implants will be in direct contact with the vessel wall we chose a direct contact multiplex assay format which allowed parallel determination of the amount of attached cells (“Viability”), the amount of dead cells (“Toxicity”) and the amount of cells undergoing apoptosis (“Apoptosis”). Cell attachment and proliferation on materials (“Viability”) was the same or only slightly reduced on film samples compared to the ideal surface of tissue culture-treated polystyrene (TCPS) (Figure 4). Since in addition, no material toxicity was observed all presented polymer films can be rated as non-cytotoxic. Also, none of the lactide/caprolactone materials led to significantly increased apoptosis induction compared to TCPS control.

#### Hemolysis

**FIGURE 5:**
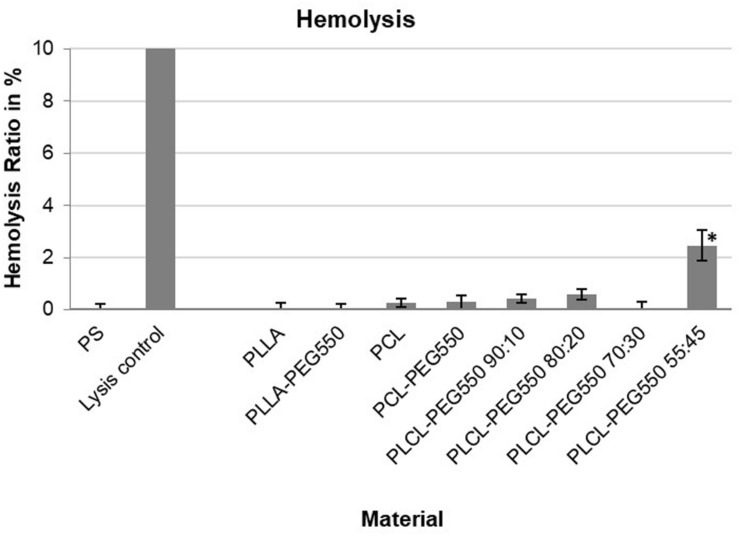
Hemolysis ratios of the tested biomaterials after 1 h of incubation with diluted whole blood at 37°C. Hemolysis of the empty well (PS) was set to 0 while lysis control equals 100%. PLCL-PEG550 55:45 exhibited significantly increased hemolysis with ^∗^*p* < 0.006 after Bonferroni correction.

Hemolysis testing is required for the evaluation of biomaterials intended for stent devices according to ISO 10993-4 (Beuth Publishing, 2017). It is a sensitive indicator of the destructive degree of a material to erythrocytes. Among the tested biomaterials only PLCL-PEG 55:45 showed an elevated hemolysis rate of 2.5% ± 0.5 (Figure 5). All other materials showed rates smaller than 2%. However, the increase in hemolysis on the surface of the material PLCL-PEG550 55:45 compared to the other materials and in relation to the threshold value of 2% was significant after Bonferroni correction (for criteria see review by Van Oeveren, 2013).

#### Platelet Adhesion and Morphology

Platelet adhesion together with morphology of adhered platelets is a common indicator to evaluate hemocompatibility of newly developed biomaterials more precisely their thrombus formation potential. Based on their shape, adhered platelets can be classified into five categories (Supplementary Table S6), whereby only category V platelets might also form aggregates and are indicative of material thrombogenicity (Ko et al., 1993).

**FIGURE 6:**
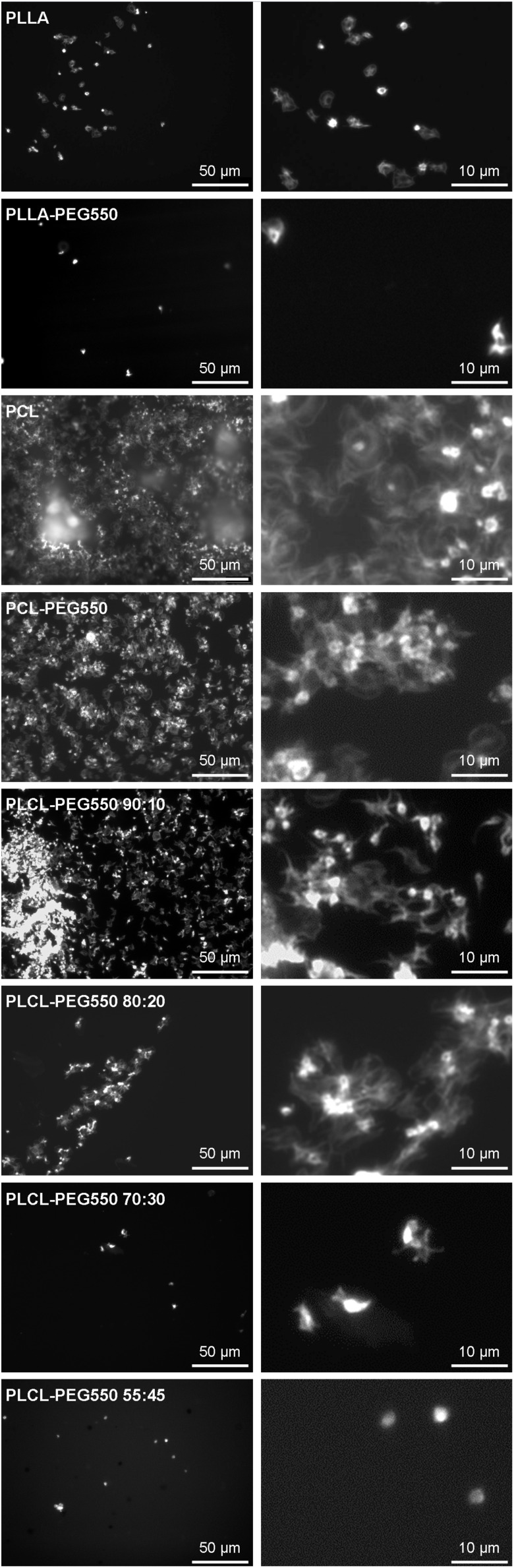
Representative micrographs of platelet adhesion onto the lactide/caprolactone polymer films. Platelet-rich plasma was incubated for 1 h on film samples and adherent platelets were visualized using *F*-actin staining. Micrographs of 500x magnification give an overview (scale bar corresponds to 50 μm) while micrographs of 2500x magnification show platelet morphology in more detail (scale bar corresponds to 10 μm).

In our study clear differences in platelet adhesion onto the various materials were observed as can be seen in the representative micrographs in Figure 6. While most materials showed low platelet adhesion and spreading, PCL control, PCL-PEG550 and PLCL-PEG550 90:10 exhibited high platelet adhesion rate accompanied by spreading to a fully spread (category V) state and formation of aggregates which indicates potential thrombogenicity of these materials. Among these, the PCL control showed most adhesion with the formation of a complete carpet of spread platelets and the presence of many aggregate buds. Onto PCL-PEG550 and PLCL-PEG550 90:10 platelet adhesion occurred in a grouped order with free spaces in between. PLLA exhibited slightly more adhered platelets than PLLA-PEG550.

#### Leucocyte Activation

**FIGURE 7:**
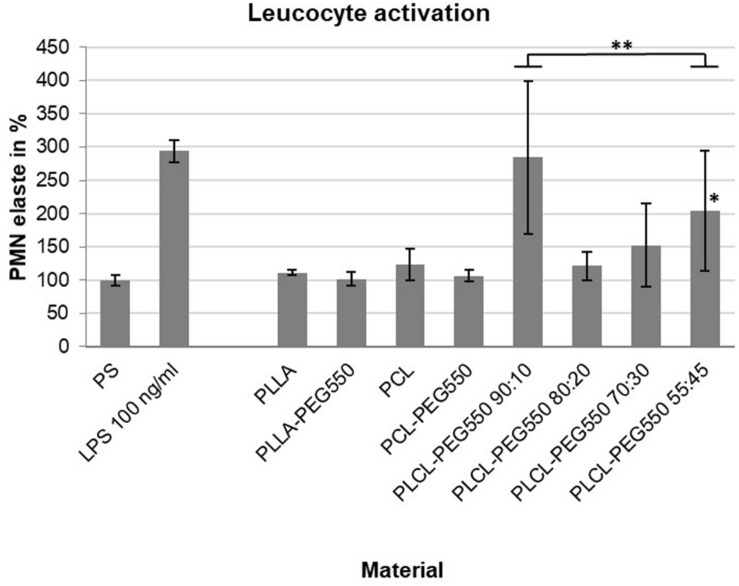
Leucocyte activation during a 1 h blood/biomaterial interaction was analyzed using PMN elastase ELISA assay. The baseline PMN elastase expression in non-activated blood in an empty PS well was set to 100%. An exemplary induction of leucocyte activation was achieved by the addition of bacterial lipopolysaccharides (LPS). Measurements were carried out in quadruplicate, ^∗^*p* ≤ 0.05; ^∗∗^*p* ≤ 0.01.

PMN elastase expression levels are an indicator for leucocyte activation since inflammatory stimulation of neutrophils – the most abundant leucocytes – results in the rapid release of high amounts of this proteolytic enzyme (Alam et al., 2012). Here, we analyzed PMN elastase release after 1 h of incubation between whole blood and material films (Figure 7). Most of the presented materials showed no significant increase in leucocyte activity. The PMN elastase value for PLCL-PEG550 90:10 was increased on average, but did not turn out to be significant compared to the control. In contrast, the value for material type PLCL-PEG550 55:45 showed a significant increase.

#### Endothelial Cell Adhesion

**FIGURE 8:**
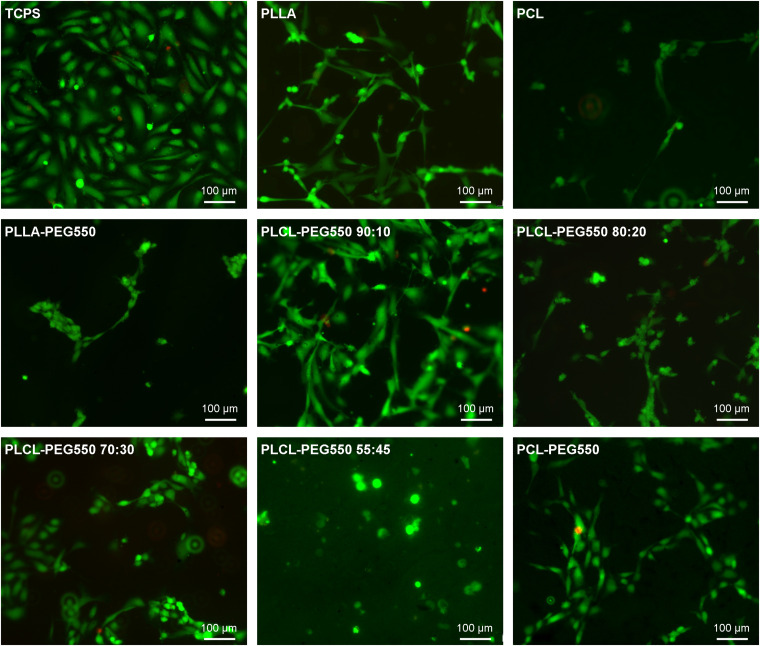
Representative micrographs of adherent endothelial cells on the different lactide/caprolactone materials stained with Live/Dead staining. The cells were seeded directly onto material films, incubated for 24 h and stained (green: cytoplasm of viable cells, red: nuclei of cells with damaged cell membrane). Bar corresponds to 100 μm.

**FIGURE 9:**
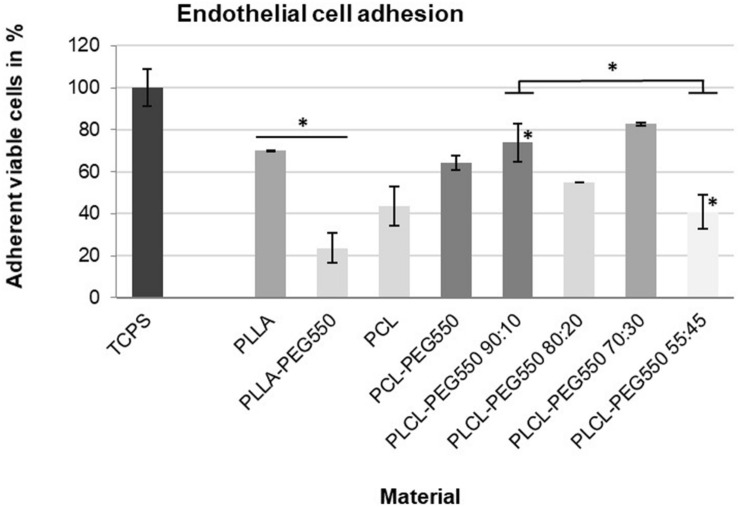
Number of viable endothelial cells attached to material disks. The cells were seeded directly onto material films, incubated for 24 h and counted after Live/Dead staining. At least 10 microscopic images were counted per material. Bar shade indicates cell morphology (from dark gray = good spreading, normal morphology to light gray = mostly non-spread); ^∗^*p* ≤ 0.01.

Since re-endothelialization is thought to greatly improve anti-thrombotic and anti-restenotic properties of a surface we analyzed each material’s potential to facilitate endothelial cell adhesion using primary human cardiac microvascular endothelial cells (HCMECs). Representative micrographs of attached HCMECs 24 h after seeding onto the different materials and stained using live/dead staining are depicted in Figure 8 and average cell numbers are given in Figure 9. Although cells on PEGylated lactide/caprolactone films were viable indicating again that the materials are non-toxic, big differences in number of attached cells and cell morphology were observed. From the described materials PCL-PEG550 and PLCL-PEG550 90:10 showed highest cell adhesion accompanied by a high grade of spreading. While on PCL-PEG550 ECs appeared in ordered groups cell adhesion in case of PLCL-PEG550 90:10 the ECs seemed more evenly distributed. Although even more endothelial cells were able to attach onto PLCL-PEG550 70:30, cells had a rounder shape and cluster formation was observed. Among the tested samples PLLA-PEG550 and PLCL-PEG550 55:45 exhibited lowest cell numbers and nearly no spreading was seen.

## Discussion

### PLCL-PEG Copolymerization Strategy Resulted in Highly Accurate Composition

Copolymerizing lactide with caprolactone and incorporating a polyethylene glycol end chain has been successfully achieved. The intended molar ratios of lactide to caprolactone (100:0, 90:10, 80:20, 70:30, 55:45, 0:100) were achieved with high accuracy to an industrial standard and within ±2 moles of the target molar fractions (Table 1). The presence of PEG (*M*_n_ = 550 Da) was confirmed using ^1^H-NMR while all synthesized polymers presented low polydispersity indexes and outstanding control. Furthermore, copolymers were synthesized with high molecular weights (*M*_w_ = 128 – 348 kDa) which remains challenging with conventional polymerization procedures (Auras et al., 2004; Lasprilla et al., 2012). Polymerization reported herein is even more impressive considering that these polymers were produced entirely in the absence of toxic organic solvents yielding cleaner biomaterials that elicit lowered inflammatory response.

### Film Surface Characteristics

Using WCA measurements, FTIR-ATR and SEM imaging we confirmed the high quality of our film preparation and analyzed the surface characteristics in more detail (see Table 2 for summary). While all films were flat without the presence of air bubbles and holes, differences in their surface morphologies and topographies were observed. Surface texturing was observed in PCL and PCL-PEG550 in form of spherulite crystallization as seen in SEM images (Figure 3) which was also confirmed by the presence of a typical band shift of the carbonyl peak in FTIR (Figure 2). This surface inhomogeneity also explains the higher variance in contact angle measurements. PLCL-PEG550 55:45 was shown to exhibit high grade of crystallinity which was represented in SEM in the formation of ridged structures and in FTIR by the existence of a lower wavelength shoulder of the carbonyl peak. These changing surface properties relate to the solvent casting process and material behavior. Since all films were cast using the same method, we believe that material behavior leads to the formation of a slightly varied surface quality. No clear trend was observed in the other PEG-modified films: While PLLA-PEG550 and PLCL-PEG550 70:30 exhibited smooth surfaces PLCL-PEG550 90:10 showed slight surface roughness and PLCL-PEG550 80:20 consisted of smooth areas and more roughened areas.

All PEG-containing materials showed lower water contact angles than the controls – although to different degrees – indicating that PEG addition increased hydrophilicity. This also proves that PEG groups were present at the surfaces of all sample films whereby hydrophilicity was highest in PCL-PEG550, PLCL-PEG550 90:10 and 80:20. The values for PEG functionalized PLCL films were significantly lower than values reported in the literature for non-PEGylated lactide-caprolactone polymers owing to the incorporation of PEG in the polymer structure (Lim et al., 2008; Inthanon et al., 2016).

### *In vitro* Biocompatibility

#### Copolymerization Method Has No Negative Effect on Cells

General cytocompatibility testing as required by ISO 10993-5 (Beuth Publishing, 2009) was conducted in direct contact format – seeding the L929 cells directly onto film samples – because the materials are intended as cardiovascular materials that will directly be colonized *in vivo*. With L929 cell line no significant differences in number of viable adherent cells were observed among all tested materials. Thus, together with the results of absolutely no toxicity, we concluded that the presented copolymerization strategy generated completely non-toxic materials. In addition, no other adverse effects as measured by apoptotic induction were observed.

Hemolysis on the one hand is a measure of blood toxicity (Beuth Publishing, 2017) for example originating from residual solvent in the polymer. On the other hand, specific material surface structures might cause blood cell rupture. Here, we found that PLCL-PEG copolymers were non-toxic to blood erythrocytes although possibly topography related slightly elevated hemolysis was observed in case of PLCL-PEG550 55:45. As seen in SEM, this material exhibited a high degree of surface irregularity in the form of ridges of approximately 1 μm diameter that seem to be able to lacerate red blood cells. PEG incorporation was shown to prevent hemolysis in a hemolytic material (Wang et al., 2010) but the ridges presented by PLCL-PEG550 55:45 seem to be too sharp so that the presence of PEG was partially overcompensated by the topographic effect. Surface patterning is a well-known and important tool that can be used to enhance biocompatibility of these materials for medical applications while here we can also see the potential for adverse impact that an uncontrolled surface may have (Pacharra et al., 2019).

#### Cell Adhesion and Activation Can Be Fine-Tuned by Polymer Composition

In addition to mandatory testing required for every material intended for implant use, we wanted to obtain more information regarding a possible use of these materials as cardiovascular implants. Since stents and vascular grafts will be in contact to the vessel wall and the blood, we chose three additional direct contact assays to analyze thrombogenic and inflammatory properties, and endothelialization.

Platelet adhesion, as an indicator of possible material thrombogenicity, in general could be reduced by the presence of PEG chains on the films compared to controls without PEG – although the extent depended on the polymer type. It has already been shown that the presence of PEG on the surface of a material leads to the formation of more inert surfaces with reduced cell adhesion capabilities for various materials (Chuang and Masters, 2009; Noel et al., 2015) while Chen et al. (2011) found that the degree of adhesion reduction might vary depending on the type of PEG. In case of PCL-PEG550 substantial platelet adhesion and spreading but less aggregation was observed than on un-PEGylated PCL. From film characterization via SEM, we know that these polymer surfaces presented ordered spherulitic structures which may have caused an enhanced affinity for platelet adhesion. In addition, the high platelet adhesion on both PCL and PCL-PEG550 might be due to the partial presence of negatively charged carboxyl groups at the film surface as was already described for carboxyl presenting surfaces (Sperling et al., 2009). In contrast, the very smooth and uncharged surface of PLLA and PLLA-PEG550 offered little attachment points for platelets while the presence of PEG further reduced platelet adhesion and spreading. This indicates that PEG chains were presented to the solid/liquid interface of this material. In case of the PLCL-PEG550 series the observed differences in platelet adhesion and spreading originated in the different morphologies. As the caprolactone content increased in the copolymer samples PLCL-PEG550 90:10 to 55:45, we observed that the platelet adhesion and spreading decreased. In all samples the content of caprolactone was low, not enough to create a negatively charged surface similar to that of the pure PCL-PEG550 which furthered platelet adhesion.

Overall, the lactide/caprolactone materials did not lead to substantial leucocyte activation as tested on the basis of PMN elastase release from neutrophils in human whole blood. In contrast, PLCL-PEG550 55:45 showed a significant increase in PMN elastase levels. In the case of PLCL-PEG550 55:45, the effect of surface irregularities, which has already been described in hemolysis, may come into play again and seems to be responsible for the slightly increased hemolysis activity. So far, the mechanism of neutrophil activation by a material surface is not clearly understood but surface chemistry and topography have been shown to influence neutrophil behavior (Tan et al., 2000; Patel et al., 2006). Thus, PLCL-PEG550 55:45 might lead to partial activation of neutrophils because of its roughened or ridged surface structures, respectively.

Since rapid and complete endothelialization is thought to greatly improve cardiovascular implant biocompatibility we also tested endothelial cell adhesion using primary human cardiac microvascular endothelial cells (HCMECs) – a cell type actually present in the coronary vasculature. We found that EC adhesion and spreading gradually increased with increasing lactide content and decreasing crystallinity as did platelet adhesion and spreading. This suggests that PEG presentation is strongly affected by polymer chain orientation and alignment. In PLCL-PEG550 90:10 it seemed that PEG was not accumulated at the film surface possibly due to the random polymer orientation of this amorphous material allowing cell adhesion. In contrast, PEG chains seemed to be evenly enriched at the surface of the highly crystalline PLCL-PEG550 55:45 resulting in a cell-repellent more inert surface. Thus, the presented copolymerization of lactide/caprolactone/PEG allows gradual tuning of cell adhesion properties. On the smoother materials with a better presentation of PEG chains to the interface (e.g., PLLA-PEG550) cell adhesion of endothelial cells and platelets was much lower.

*In vivo*, a fast and complete endothelialization might greatly influence the hemocompatibility of materials. On the one hand, the presence of an endothelial cell layer can cover the foreign surface from direct contact with blood cells and thereby hinder blood cell activation. In addition, healthy endothelial cells can actively prevent thrombus formation and inflammatory responses (de Mel et al., 2008; Ren et al., 2015). Therefore, PCL-PEG550, PLCL-PEG550 90:10, and PLCL-PEG550 70:30, which showed good endothelial cell coverage, might be promising candidates for cardiovascular implant production. This will be studied in more detail in the future by more advanced *in vitro* testing (for example in a bioreactor under flow conditions) or in animal experiments.

## Experimental Section

### Materials

All customized polymer formulations (PLLA-PEG550, PLCL9010-PEG550, PLCL8020-PEG550, PLCL7030-PEG550, PLCL5545-PEG550, PCL-PEG550) were supplied by Ashland Specialties Ireland Ltd. (Dublin, Ireland). PL38 and PC12 with inherent viscosity of 3.8 and 1.2 dl/g respectively were used as controls and supplied from Purac (Gorinchem, Netherlands). Chloroform (CHCL_3_ and CDCL_3_) were supplied from Sigma Aldrich and used for all film casting, GPC and NMR testing.

### Polymer Synthesis

PEG-functionalized PCL, PLLA and PLCL of varied composition were synthesized by Ashland Specialties Ireland Ltd. Briefly, these polymers were achieved through the Ring-Opening Polymerization (ROP) of the monomers L-lactide and ε-caprolactone using the catalyst stannous octoate initiated by the hydroxyl terminal group of the relevant initiation molecule (polyethylene glycol methyl ether). This resulted in polymers consisting of long chains of PLLA, PCL or PLCL connected to a singular PEG end group. In each polymerization reaction, pre-determined amounts of monomer, initiator and catalyst were added to the autoclave and were then heated to the reaction conditions. This reaction achieved good reaction control in terms of target molar mass, monomer conversion and a relatively low polydispersity polymer. A structural overview on each reaction is presented in Figure 1 and a summary of all materials used in this study is given in Supplementary Table S1.

### Characterization of Polymer Composition

Gel permeation chromatography (GPC) measurements were performed using an Agilent triple detector system which was calibrated with polystyrene standards at a concentration of 10 mg/ml. Chloroform (CHCL_3_) was used as mobile phase with a flow rate of 1.0 ml/min. Weight average molecular weight (M_w_), number average molecular weight (M_n_) and polydispersity (PDi) data were collected using refractive index peak height in the range of <10 mV. For each polymer 3–7 mg powder was dissolved in 2 ml chloroform and filtered [0.2 μm pore size, 13 mm diameter, Millipore SLFG013NL, Fluropore PTFE (F) membrane]. An Agilent Technologies column (PLgel, 5 μm MIXED-C, 300 × 7.5 mm) was used for GPC analysis.

Proton nuclear magnetic resonance spectroscopy (^1^H-NMR) was utilized for identification of the polymer, confirmation of the polymer purity in terms of monomer conversion, molar ratio of relevant copolymer units and to confirm the presence of PEG in the chemical structure. A 300 MHz Bruker NMR machine was used to obtain the spectra which were analyzed and processed using MestReC software package. Deuterated chloroform (CDCl_3_) was used to dissolve the polymer which was then filtered using a Milnex (0.2 μm) into NMR sample tubes. Lactide methyl presented a signal centered around 5.16 ppm and caprolactone on 4.02 ppm while PEG presence was identified at a chemical shift of 3.65 ppm (Wu et al., 2010). These signals were taken as reference points for the determination of the polymer composition given in Table 2. From the ^1^H-NMR we observed the polymer conversion based on the CH_3_ and CH peaks for respective polymers and monomers introduced.

### Polymer Film Preparation

Film casting was achieved by dissolving 0.5 g of polymer in 15 ml of chloroform. Each solution was dissolved until transparency and poured into a glass petri dish of dimensions 60 mm × 15 mm. Chloroform was evaporated overnight resulting in thin uniform films which were peeled from the glass surface once solvents had evaporated entirely.

### Analysis of Film Surface Properties

Water contact angle was measured to analyze the wettability of the film samples. This study was carried out using KSV Cam 200 optical goniometer (Helsinki, Finland). Hundred μl of deionized water was dropped on the surface of the films using a micro-syringe. Images were captured with a frame interval of 1 s. KSV Cam software was used to analyze the images. This was done at Eastman Dental Institute, University College London, United Kingdom. For each material, the contact angles of three independent samples were measured with at least 10 measurements per sample.

Fourier transform infrared (FTIR) spectroscopy was performed as attenuated total reflectance (ATR) measurement. Spectral manipulations were performed by spectroscopy software OPUS provided by Bruker.

The ATR measurements were performed by a Tensor 27 FTIR spectrometer from Bruker, equipped with a VariGATRTM ATR accessory, with a resolution of 4 cm^–1^, for each sample using 50 scans. The spectra were acquired by a liquid N_2_ cooled mercury cadmium telluride (MCT) detector, in the range of 4000–600 cm^–1^.

Scanning electron microscopy (SEM) images were obtained using a Quanta 3D FEG scanning electron microscope (FEI). Samples were cut from the central part of the produced films and were fixed on a commercial aluminum stub with conductive carbon tape (Plano), in a way that a part of the film sample was overlapping and no contact to the stub or carbon tape occurred. This part of the sample has always been analyzed. The optimum in image quality was obtained with an acceleration voltage of 2 kV, a spot size of 4.5 and a working distance of around 10 mm. Under these conditions no or a minimum of degradation of the sample during imaging was observed.

### Toxicity Testing With L929 (Multiplex Assay)

The mouse fibroblast cell line L929 was obtained from DSMZ (Leibniz-Institut DSMZ - Deutsche Sammlung von Mikroorganismen und Zellkulturen GmbH) and handled according to their manual. Briefly, L929 cells were grown in RPMI 1640 medium (PAN Biotech, Aidenbach, Germany) supplemented with 10% FBS (PAN Biotech) and a penicillin/streptomycin mixture (Thermo Fisher Scientific, Dreieich, Germany) in an incubator (37°C, 5% CO_2_). For subculturing cells were detached using 0.25% Trypsin solution (Thermo Fisher Scientific).

This multiplexed assay was adapted from Neuss et al. (2008). From each polymer film disks of 6 mm diameter were generated by punching. Disinfection was achieved by incubation in 70% ethanol followed by washing with sterile water and equilibration in medium. Material disks of 6 mm in diameter were placed into the wells of a 96 well polystyrene(PS) plate and fixed with Teflon rings. Tissue culture-treated polystyrene (TCPS, Nunc) plates were used as controls for ideal cell growth. L929 cells (2⋅10^4^ cells per well) were seeded on top of the disks and grown for 24 h under standard cell culture conditions. Blank values were obtained from wells without material disks and cells. As a positive control for cytotoxicity cells grown on TCPS were lysed prior to assaying. As a positive control for apoptosis 100 nM staurosporine (Santa Cruz Biotechnology, Heidelberg, Germany) was added to cells grown on TCPS 6 h after seeding. Each sample was analyzed in quadruplicate.

After 24 h of incubation the cell culture supernatants were transferred into black 96 well plates and subjected to cytotoxicity analysis using the CytoTox-ONE^TM^ Homogeneous Membrane Integrity Assay (Promega, Mannheim, Germany) following the manufacturer’s protocol. After equilibration to ambient temperature assay reagent was added to each well in a 1:1 ratio and incubated for 10 min. The reaction was stopped by addition of Stop Solution and the fluorescence monitored (excitation: 560 nm, emission: 590 nm).

Directly after supernatant removal for cytotoxicity analysis cell viability was analyzed using the CellTiter-Blue^®^ Cell Viability Assay (Promega) following the manufacturer’s protocol. After addition of the reagent/medium mixture plates were incubated for 2 h at 37°C, 5% CO_2_. Cell culture supernatant was transferred to black 96 well plates and fluorescence monitored (excitation: 560 nm, emission: 590 nm).

Directly after supernatant removal for viability measurement cells were subjected to apoptosis analysis using the Apo-ONE^®^ Homogeneous Caspase-3/7 Assay (Promega) following the manufacturer’s protocol. After addition of the reagent/medium mixture plates were incubated for 2 h at ambient temperature. The supernatant was transferred to black 96 well plates, incubated for further 18 h and the fluorescence monitored (excitation: 485 nm, emission: 527 nm).

### Blood Collection

Fresh human whole blood was obtained from healthy adult volunteers (no medication (e.g., aspirin) in the previous 2 weeks) by venipuncture into either *S*-Monovette 9 ml LH (Sarstedt, Nümbrecht, Germany). Blood was collected at the Centre for Translational Medicine, Medical Department I, University Hospital of the Ruhr-University Bochum, Herne, Germany according to appropriate legal and ethical guidelines. The participants provided written informed consent to participate in this study. The study was reviewed and approved by the Ethics Committee of the Medical Faculty of the Ruhr-University Bochum, Gesundheitscampus 33, D-44801 Bochum. The registration number of the study is 16-5649.

### Hemolysis

Hemolysis testing was done based on the protocol by Wang et al. (2013) with slight modifications. Lithium heparin (LH) blood was diluted 4:5 in 0.9% (w/v) sodium chloride (NaCl) solution. Material disks of 14 mm diameter were placed into the wells of a PS 24 well plate (Nunc, Fisher Scientific, Schwerte, Germany) and fixed using silicone rings. After washing with 0.9% NaCl solution the disks were incubated for 30 min at 37°C with 980 μl NaCl solution. As negative control the bare PS was used and as positive control of complete red blood cell lysis 980 μl of distilled water was added instead of 0.9% NaCl. To each well 20 μl of pre-diluted blood was added, mixed and incubated for 60 min at 37°C with slow shaking. After centrifugation at 700 × *g* for 10 min 200 μl of supernatant were transferred to a 96 well plate and the absorbance was measured at 542 nm using 691 nm as reference wavelength. Measurements were done in quadruplicate. The hemolysis ratio (HR, in %) was calculated as follows *HR = (A – C_neg)_ / (C_pos_ – C_neg_)* x 100%, where A is the absorbance of the sample, C_neg_ is the absorbance of the negative control and C_pos_ is the absorbance of the positive control.

### Platelet Adhesion and Morphology

Platelet-rich plasma (PRP) was isolated from LH blood according to the description from Parnham and Wetzig (Parnham and Wetzig, 1993). Briefly, whole blood was centrifuged at 250 × *g* for 10 min and the supernatant (=PRP) was recovered.

Material disks of 14 mm diameter were placed into the wells of a 24 PS well plate and fixed using silicone rings. After washing with 0.9% NaCl solution three times the disks were incubated at 37°C for 1 h with 500 μl of PRP. Adhered platelets were visualized using F-actin staining. Briefly, disks were washed three times with PBS (PAN-Biotech), fixed with 4% formaldehyde (Life Technologies, Darmstadt, Germany) in PBS, permeabilized with 0.4% Triton X-100 in PBS and blocked using 2% BSA in PBS. After staining with PromoFluor 546 phalloidin (PromoKine) the disks were mounted using Fluoroshield with PPD. Fluorescence microscopy was done with the IX 51 microscope (Olympus, Hamburg, Germany). At least 2 independent samples were visualized per material and at least 5 images of different areas per sample were recorded.

### Leucocyte Activation

Material disks of 14 mm diameter were placed into the wells of a PS 24 well plate, fixed using silicone rings and disinfected in 70% ethanol. After washing with 0.9% NaCl solution the disks were incubated at 37°C for 1 h with 500 μl of LH blood. 100 ng/ml lipopolysaccharides (LPS) from *E. coli* (Sigma-Aldrich, Schnelldorf, Germany) were used as positive control. From each sample, the plasma was isolated by centrifugation at 1400 × g for 15 min.

Leucocyte activation of these plasma samples was then assessed based on expression analysis of polymorphonuclear neutrophil (PMN) elastase using the PMN elastase ELISA (Demeditec, Kiel, Germany) according to the manufacturer’s protocol. Measurements were carried out in quadruplicate. The concentration of PMN elastase was determined from absorbance measurements by using the 4-parameter algorithm.

### Endothelial Cell Adhesion

The primary human cardiac microvascular endothelial cells (HCMEC) were obtained from PromoCell (Heidelberg, Germany) and cultured according to the manufacturer’s protocol in endothelial cell growth medium MV. Medium was changed every 2–3 days while subculturing was performed at 70–90% confluence using the detach kit from PromoCell.

The polymer films disks of 14 mm diameter were generated by punching. Disinfection was achieved by incubation in 70% ethanol followed by washing with sterile water and equilibration in medium. Material disks were placed into the wells of a 24 well PS plate and fixed with Teflon rings. Cell-culture treated polystyrene (TCPS) plates were used as controls for ideal cell growth. Cells were seeded on top of the disks (5⋅10^4^ cells per disk) and grown for 24 h under standard cell culture conditions. Staining was done using the Live/Dead Cell Staining Kit II (PromoKine, Heidelberg, Germany) followed by fluorescence microscopy using the IX 51 microscope. At least 2 independent samples were visualized per material and at least 5 images of different areas per sample were recorded and counted.

### Statistical Analysis

Values are given as mean together with the standard deviation (number of replicates is given in each experimental section). Statistical significance was determined using independent sample *t*-tests. The Bonferroni method was used to adjust for multiple comparisons (Noble, 2009).

## Conclusion

A logical series of new copolymers have been synthesized for application in a fully resorbable cardiovascular medical device consisting of a bulk polymer of lactide and caprolactone in systematic fractions and a polyethylene glycol end functional group. Poly-(L-lactide-co-ε-caprolactone) with lactide: caprolactone contents of 100:0, 90:10, 80:20, 70:30, 55:45, 0:100 have been successfully synthesized and evaluated. Polymer chemical properties were well controlled with respect to molar fractions, polymer purity, molar mass, PEG presence and polydispersity. Excellent control over material chemistry yielded a systematic spectrum of materials with tailored copolymer fractions and thereby allowed us to make comparisons between subsets of chemical compositions and to draw relevant conclusions.

Biological evaluation of material films revealed that all polymers were non-toxic, non-apoptotic and did not induce significant hemolysis while only the polymer PLCL 55:45-PEG550 induced a mild hemolytic and leucocyte (PMN elastase) response. This work indicated again that polymer chemistry is only one angle while surface morphology had a significant role to plan. From a hemocompatibility perspective, PLLA-PEG550 and PLCL-PEG550 70:30 (non-toxic, non-hemolytic, non-inflammatory and non-thrombogenic) presented the best candidates for use in cardiovascular implants while from a endothelialization perspective, PCL-PEG550, PLCL-PEG550 90:10, and PLCL-PEG550 70:30 showed best endothelial cell adhesion. In light of the overall application target, it might actually be more important in the *in vivo* situation to execute this improved endothelial cell adhesion over platelet repelling properties since a fully endothelialised surface is considered to offer ideal hemocompatibility.

## Data Availability Statement

All datasets generated for this study are included in the article/Supplementary Material.

## Ethics Statement

The studies involving human participants were reviewed and approved by the Ethics Committee of the Medical Faculty of the Ruhr-University Bochum, Gesundheitscampus 33, D-44801 Bochum. The patients/participants provided their written informed consent to participate in this study.

## Author Contributions

PD supported the polymer synthesis and characterisation. PB carried out the WCA measurements. WY performed the FTIR-ATR analysis of copolymers and controls. SS executed all SEM experiments and supported the image interpretation. US and NB were responsible for the acquisition of the donors, their education, the proper blood collection, and the immediate transport of the fresh blood samples to the laboratory. IR was involved in WCA analytics and critical proofreading. RV carried out a critical proofreading as well. WW was responsible for the concept of copolymer synthesis and functionalization and supervised SM and PD. JS was responsible for the overall design of this research work, supervised particularly SP and was involved in all biological work of this manuscript. All the authors contributed to the article and approved the submitted version.

## Conflict of Interest

SM and PD were employed by the company Ashland Specialties Ireland.

The remaining authors declare that the research was conducted in the absence of any commercial or financial relationships that could be construed as a potential conflict of interest.
