# Supplementary material for: Cytocompatibility Evaluation of a Novel Series of PEG-Functionalized Lactide-Caprolactone Copolymer Biomaterials for Cardiovascular Applications
Source: Front Bioeng Biotechnol. 2020 Aug 13;8:991. doi: 10.3389/fbioe.2020.00991 (PMC7438451; doi:10.3389/fbioe.2020.00991)
Supplement: Supplementary file 1 [file Data_Sheet_1.pdf]

Supplementary Material

1 Supplementary Data

Supplementary information to 2.1.1 Synthesis:

Table S1. Abbreviations and full chemical names of all lactide and caprolactone polymers used in this study.

Material Family	Chemical Name	Abbreviation
PLLA	Poly(L-lactide)	PLLA (PL38) Control
	Poly(L-lactide)-co-polyethylene glycol	PLLA-PEG550
PCL	Polycaprolactone	PCL (PC12) Control
	Polycaprolactone-co-polyethylene glycol	PCL-PEG550
PLCL	Poly(L-lactide-co-ε-caprolactone) (90:10)-co-polyethylene glycol	PLCL-PEG550 90:10
	Poly(L-lactide-co-ε-caprolactone) (80:20)-co-polyethylene glycol	PLCL-PEG550 80:20
	Poly (L-lactide-co-ε-caprolactone) (70:30)-co-polyethylene glycol	PLCL-PEG550 70:30
	Poly (L-lactide-co-ε-caprolactone) (55:45)-co-polyethylene glycol	PLCL-PEG550 55:45

Supplementary information to 2.1.2 Surface Properties of Polymer Films:

Table S3. Characteristic infrared bands of PCL (PC12)

Position /cm^{-1}	Vibrational Mode	Abbreviation
1162	stretching	$\nu_{amorphous}$
1180	sym. stretching	ν_s (C-O-C)
1192	stretching	ν (OC-O)
1243	asym. stretching	ν_{as} (C-O-C)
1294	stretching	$\nu_{crystalline}$
1726	stretching	ν(C=O)
2864	sym. stretching	ν_s (CH_2)
2946	asym. stretching	ν_{as} (CH_2)

Table S4. Characteristic infrared bands of PLA (PL38)

Position /cm^{-1}	Vibrational Mode	Abbreviation
868	stretching	ν_{as} (C-COO)
1045	sym. stretching	ν_s (C-CH$_3$)
1086	sym. stretching	ν_s (C-O-C)
1182	asym. stretching + twisting	ν_s (C-O) + τ (CH$_3$)
1360	sym. Scissoring	δ_s (CH$_3$)
1450	asym. scissoring	δ_{as} (CH$_3$)
1755	Stretching	ν(C=O)
2943	sym. Stretching	ν_s (CH$_2$)
2993	asym. stretching	ν_{as} (CH$_2$)

Table S5. Characteristic infrared bands of PEG550

Position /cm^{-1}	Vibrational Mode	Abbreviation
843	rocking	ρ (CH$_2$)
963	rocking	ρ (CH$_2$)
1062	stretching	ν (CO)
1110	stretching	ν (CO)
1150	stretching	ν (CO)
1242	twisting	τ (CH$_2$)
1281	twisting	τ (CH$_2$)
1343	wagging	ω (CH$_2$)
1361	wagging	ω (CH$_2$)
1468	scissoring	δ (CH$_2$)
2886	sym. stretching	ν_s (CH$_2$)
2946	asym. stretching	ν_{as} (CH$_2$)

2 Supplementary Figures

Supplementary information to 2.1.2 Surface Properties of Polymer Films:

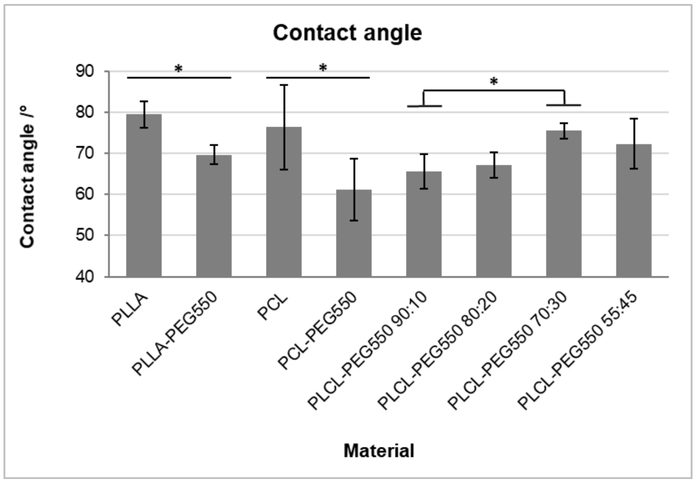

Supplementary Figure S2: **Supplementary Figure S2**: Water contact angles of the materials. Error bars represent standard deviation. *p* statistical significance values ($^{*}p \leq 0.01$) were determined for PLLA vs. PLLA-PEG550, PCL vs. PCL-PEG550 and PLCL-PEG550 90:10 vs. PLCL-PEG550 70:30 (n = 3).

Supplementary information to 2.2.3. Platelet Adhesion and Morphology:

Table S6. Five categories of platelet morphology upon adhesion to foreign surfaces according to Ko et al. (Ko et al., 1993).

Category	Name	Morphology
I	Round	Round or discoid with no pseudopodia
II	Dendritic	Early pseudopodial, no flattening evident
III	Spread-dendritic	Intermediate pseudopodial, some pseudopodia flattened
IV	Spreading	Late pseudopodial, hyaloplasm spreading
V	Fully spread	Hyaloplasm well spread, no distinct pseudopodia
