# Supplementary figures and images for: Cytocompatibility Evaluation of a Novel Series of PEG-Functionalized Lactide-Caprolactone Copolymer Biomaterials for Cardiovascular Applications
Source: Front Bioeng Biotechnol. 2020 Aug 13;8:991. doi: 10.3389/fbioe.2020.00991 (PMC7438451; doi:10.3389/fbioe.2020.00991)

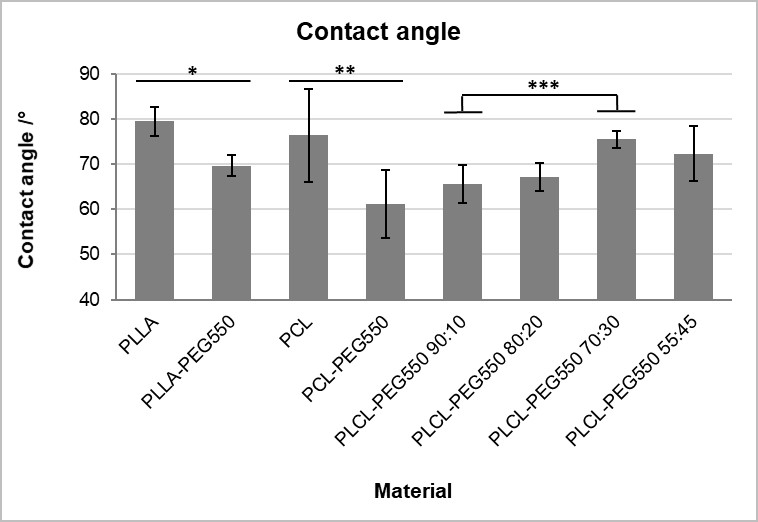

Supplement: Supplementary file 2 [file Image_1.JPEG]
